# Animal Models of Steroid-Induced Osteonecrosis of the Femoral Head Established by Different Doses of Lipopolysaccharide Combined with Glucocorticoids: A Systematic Review and Network Meta-Analysis

**DOI:** 10.3390/ijms27125416

**Published:** 2026-06-16

**Authors:** Wenlong Luo, Yuchen Duan, Haoyun Huang, Xinyi Li, Yi Zhou, Yimei Hu

**Affiliations:** Clinical Medical College, Chengdu University of Traditional Chinese Medicine, Chengdu 610072, China; luowenlong@stu.cdutcm.edu.cn (W.L.); duanyuchen6334@163.com (Y.D.); huanghy0531@stu.cdutcm.edu.cn (H.H.); lililixxxy@163.com (X.L.)

**Keywords:** lipopolysaccharide, glucocorticoid, steroid-induced osteonecrosis of the femoral head, TLR4/NF-κB, osteocyte apoptosis, endothelial dysfunction, oxidative stress, angiogenesis, network meta-analysis, molecular mechanism

## Abstract

Steroid-induced osteonecrosis of the femoral head (SONFH) is commonly modeled using lipopolysaccharide (LPS) combined with glucocorticoids, but the comparative performance of different LPS doses remains unclear. We conducted a network meta-analysis following PRISMA guidelines and registration in PROSPERO (CRD420261346269) to compare different LPS dose regimens combined with glucocorticoids for establishing SONFH animal models. Embase, PubMed, and Web of Science were searched through December 2025 for randomized controlled animal studies. The primary outcome was the empty osteocyte lacunae rate, and the secondary outcome was the modeling success rate. Forty articles comprising 49 studies and 923 animals were included. Compared with normal controls, low-, medium-, and high-dose LPS combined with glucocorticoids all significantly increased the empty osteocyte lacunae rate and modeling success rate. High-dose LPS produced a significantly greater empty lacunae rate than low-dose LPS, whereas most other pairwise comparisons were not statistically significant. Subgroup analyses indicated that modeling efficacy was influenced by animal species, glucocorticoid dose, and modeling duration, while high-dose LPS may be associated with increased mortality risk. Overall, LPS plus glucocorticoids effectively induces SONFH-like pathological changes, but protocol selection should balance modeling efficacy and safety, and no single definitively optimal regimen can yet be concluded.

## 1. Introduction

Steroid-induced osteonecrosis of the femoral head (SONFH) is a refractory orthopedic disorder induced by long-term or high-dose glucocorticoid (GC) administration [1,2]. It is characterized by impaired blood supply to the femoral head, osteocyte necrosis, structural destruction, and abnormal joint function, which can lead to disability in advanced stages [3]. Clinically, it manifests mainly as hip pain and limited joint mobility. The incidence of SONFH is increasing annually in China and tends to affect younger patients. With the expanded clinical use of glucocorticoids, the number of affected individuals continues to rise, imposing a heavy burden on patients and social medical resources [4]. At the molecular level, SONFH is associated with glucocorticoid-triggered osteocyte apoptosis, suppression of osteoblast differentiation, lipid deposition, oxidative stress, endothelial dysfunction, and microthrombus formation, which ultimately compromise femoral head perfusion and bone remodeling. Therefore, the establishment of highly stable and reproducible animal models is a core prerequisite for in-depth exploration of the pathogenesis of SONFH and the development of novel preventive and therapeutic strategies. The quality of animal modeling directly affects the scientific validity and reliability of subsequent research outcomes.

Currently, the main induction methods for SONFH animal models include glucocorticoids alone, glucocorticoids combined with serum, and glucocorticoids combined with lipopolysaccharide (LPS). The glucocorticoid-only method is simple to perform but requires a long modeling period and achieves a low success rate. Other combined approaches, such as glucocorticoids plus horse serum, are limited by complicated procedures, high costs, and restricted applicability [5]. In contrast, the LPS-plus-glucocorticoid protocol has been widely adopted in SONFH-related experimental research because of its short modeling cycle, high success rate, and ability to mimic the clinical pathogenic scenario of glucocorticoid exposure combined with inflammatory stress [6]. From a molecular perspective, LPS can activate innate immune signaling and downstream pro-inflammatory cytokines, aggravate endothelial injury, and promote coagulation abnormalities, whereas glucocorticoids amplify osteocyte death, adipogenic shift in marrow stromal cells, and vascular rarefaction; together these processes reproduce several key pathogenic axes implicated in SONFH. Despite its widespread use, considerable variation exists among studies in terms of LPS dosage, glucocorticoid dosage, modeling duration, and animal species, resulting in inconsistent modeling efficacy and safety. Furthermore, divergent evaluation criteria for modeling stability, pathological features, and clinical relevance further increase heterogeneity among studies and hinder reliable comparisons. Although several systematic reviews and meta-analyses on SONFH animal models have been published in recent years, most have employed conventional meta-analysis, which allows only pairwise comparisons of interventions and cannot achieve simultaneous ranking and comprehensive evaluation of multiple modeling protocols [7]. In addition, few studies have systematically stratified LPS into low-, moderate-, and high-dose groups to clarify dose–effect relationships. Key influencing factors—including animal species, modeling duration, glucocorticoid dosage, and sex—have not been integrated into a unified framework for multidimensional analysis. Moreover, there is a lack of balanced assessments between modeling efficacy and animal mortality, making it difficult to establish standardized protocols that can directly guide experimental practice.

Network meta-analysis enables the simultaneous comparison of multiple intervention strategies within a unified evidence framework, thereby overcoming the limitations of conventional meta-analysis, which is restricted to direct pairwise comparisons and cannot adequately incorporate indirect evidence across multiple protocols. This approach allows a more comprehensive and precise evaluation of the modeling value of different LPS–plus–glucocorticoid regimens and is particularly well suited for exploring dose–effect relationships [8]. Accordingly, the present study employed network meta-analysis to systematically assess the modeling efficacy of LPS combined with glucocorticoids at various dosages for the induction of SONFH and to clarify the key influencing factors and their regulatory patterns. This work aims to provide high-quality evidence-based support for the standardized establishment of SONFH animal models, addressing the deficiencies of existing research in the optimal selection of multiple protocols and integrated multi-factor analysis.

## 2. Methods

The methodology of this study adheres to the Preferred Reporting Items for Systematic Reviews and Meta-Analyses (PRISMA) guidelines [9].

### 2.1. Literature Search

We performed electronic searches in the following databases: Embase, PubMed, and Web of Science. No language restrictions were applied, and studies published through December 2025 were retrieved, with search strategies tailored to the specific requirements of each database. For instance, the search string used in Web of Science was as follows: TS = ((osteonecrosis of the femoral head OR femoral head osteonecrosis) AND (glucocorticoid OR steroid OR hormone) AND (rabbit OR rat OR mouse)).

### 2.2. Inclusion Criteria

Animal studies meeting the following criteria were included in this research:

- The experimental group received LPS combined with glucocorticoids for model establishment, while the corresponding control group was administered normal saline;
- The animals were rabbits, rats, or mice;
- An animal model for steroid-induced osteonecrosis of the femoral head was established;
- The study was a randomized controlled animal experiment.

### 2.3. Exclusion Criteria

Studies meeting the following criteria were excluded from the analysis:

- In vitro studies, case reports, clinical trials, reviews, abstracts, comments, and editorials;
- Studies without a normal control group;
- Duplicate publications;
- Studies involving non-steroid-induced osteonecrosis of the femoral head.

### 2.4. Outcome Measures

The primary outcome was the empty osteocyte lacunae rate in the femoral head, defined as the proportion or percentage of osteocyte lacunae without visible osteocyte nuclei on histopathological examination of femoral head sections. This parameter was extracted as reported in the original studies and was used as a quantitative indicator of osteocyte death and osteonecrotic damage. Because histological evidence of empty lacunae is a commonly used pathological hallmark of steroid-induced osteonecrosis of the femoral head, it was selected as the primary outcome in this meta-analysis [10].

The secondary outcome was the modeling success rate. Given the variability in diagnostic criteria across animal studies, we did not re-define model success using a unified threshold. Instead, the modeling success rate was recorded strictly according to the definition provided in each original study and analyzed as a binary outcome (success/failure). In other words, animals classified by the original authors as having successfully developed steroid-induced osteonecrosis of the femoral head were counted as “success,” whereas those not meeting the authors’ criteria were counted as “failure.” This approach was adopted to preserve the original study-level definitions and maximize comparability across the included experiments [5]. Because diagnostic criteria for modeling success varied across studies, including differences in histopathological thresholds, empty lacunae assessment, and imaging-based criteria, we extracted the success rate according to each original study definition rather than imposing a post hoc unified threshold. This approach avoided arbitrary reclassification of animals when individual-level diagnostic data were unavailable. The potential influence of heterogeneous definitions was further evaluated using sensitivity analysis.

### 2.5. Selection of Studies

After excluding duplicate reports, we independently evaluated the titles and abstracts of the remaining articles to exclude studies that did not meet the inclusion criteria. We subsequently reviewed the full texts of the remaining eligible articles to confirm their final inclusion. Any disagreements among the authors during the screening process were resolved through discussion.

### 2.6. Risk of Bias Assessment

We independently assessed the risk of bias using the SYRCLE tool [11], which consists of 10 items across 6 domains: ① selection bias, ② performance bias, ③ detection bias, ④ attrition bias, ⑤ reporting bias, and ⑥ other sources of bias. Studies meeting the predefined criteria were classified as having a low risk of bias, while those failing to meet the criteria were rated as having a high risk. Studies with unclear descriptions of bias were categorized as having an uncertain risk of bias. Any disagreements were resolved through consensus discussion.

### 2.7. Data Extraction

Data extraction was performed by two independent reviewers and verified by a third reviewer. The following key information was extracted from each included study: author name, year of publication, animal species, age, sex, sample size, modeling protocol for inducing steroid-induced osteonecrosis of the femoral head, interventions administered in the control and experimental groups, and primary and secondary outcome measures. For continuous variables, the means and standard deviations (SD) were extracted.

### 2.8. Data Analysis

The mean values and standard deviations of continuous variables were entered into Microsoft Excel for data organization. Statistical analysis and graphical visualization were performed using Review Manager 5.4 and Stata 18.0 software. Between-study heterogeneity was evaluated using the I^2^ statistic, with I^2^ > 50% considered indicative of significant heterogeneity; in such cases, a random-effects model was adopted. The present study was conducted in accordance with the PRISMA statement and referred to the PRISMA-NMA extension for reporting network meta-analysis. Network meta-analysis was performed within a frequentist framework, as the evidence network was relatively simple and the data structure and sample size were moderate. This approach provides standard and easily interpretable effect estimates, including mean differences (MD), relative risk (RR), and 95% confidence intervals (CI), with good reproducibility. A random-effects consistency model was applied because the network was star-shaped, with the normal control group serving as the common comparator, and no closed loops were formed among the LPS dose groups. Therefore, loop-specific local inconsistency testing was not applicable, and based on the network structure, the consistency model was considered appropriate. The empty lacunae rate was analyzed using MD with CI, and the modeling success rate was assessed using relative risk RR with 95% CI. The surface under the cumulative ranking curve (SUCRA) procedure was applied to rank the three modeling methods. Meta-regression was conducted to explore the effects of animal species and glucocorticoid dosage on heterogeneity. Subgroup analyses were performed according to animal species, glucocorticoid dosage, sex, and modeling duration. Sensitivity analysis using the leave-one-out method was conducted to verify the robustness of the results. Publication bias was visually assessed using funnel plots. A *p* < 0.05 was considered statistically significant.

## 3. Results

### 3.1. Literature Selection

**Figure 1:**
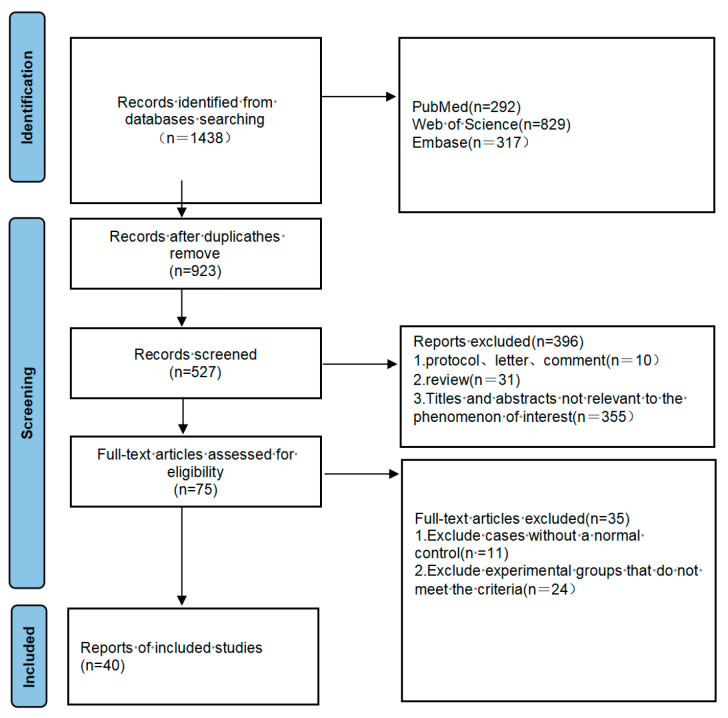
Flow chart of literature screening.

A total of 1438 articles were retrieved from three English databases, among which 515 were excluded because of duplicate entries. Following the initial screening (including review of titles, abstracts, and document types), 923 studies were retained, while an additional 396 studies were excluded because they corresponded to academic dissertations, conference proceedings, research reports, literature reviews, or in vitro studies. In the second screening phase, the full texts of the remaining 527 studies were evaluated. After studies with available outcome data were identified, 75 studies remained, and 35 were excluded because of the absence of normal control groups or noncompliance with the predefined study design criteria. Ultimately, 40 studies were selected for inclusion in this meta-analysis [12,13,14,15,16,17,18,19,20,21,22,23,24,25,26,27,28,29,30,31,32,33,34,35,36,37,38,39,40,41,42,43,44,45,46,47,48,49,50,51]. The study selection process is illustrated in Figure 1.

### 3.2. Research Characteristics

**Figure 2:**
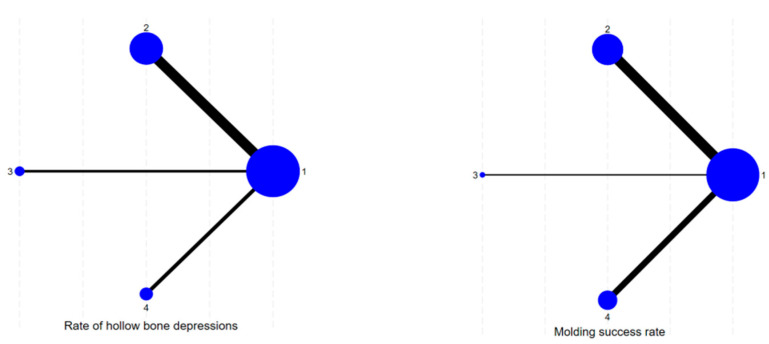
Comparative network of Bayesian network meta-analysis. Note: 1 represents the normal control group; 2 represents the low-dose lipopolysaccharide group; 3 represents the medium-dose lipopolysaccharide group; 4 represents the high-dose lipopolysaccharide group. The same applies to the following figures.

This analysis included 40 publications. Owing to variations in modeling duration across some studies, a total of 49 independent comparisons were derived, covering 923 animals. In the experimental groups, steroid-induced osteonecrosis of the femoral head was established by lipopolysaccharide combined with glucocorticoid administration. On the basis of the total dosage, the cells were stratified into low-dose (<100 μg/kg), medium-dose (100 μg/kg ≤ LPS < 1000 μg/kg), and high-dose (≥1000 μg/kg) subgroups. The primary outcome measure of the included studies was the empty lacunae rate of the femoral head [12,13,15,16,17,19,20,21,23,29,30,31,34,36,37,38,39,44,45,46,48,49,50], whereas the success rate of model establishment served as the secondary outcome measure [14,15,16,18,21,22,24,25,26,27,28,32,33,34,35,40,41,42,43,44,46,47,49,50,51]. The intervention duration ranged from 2 to 16 weeks. All the comparative results from the network meta-analysis are presented in Figure 2.

### 3.3. Risk of Bias

**Figure 3:**
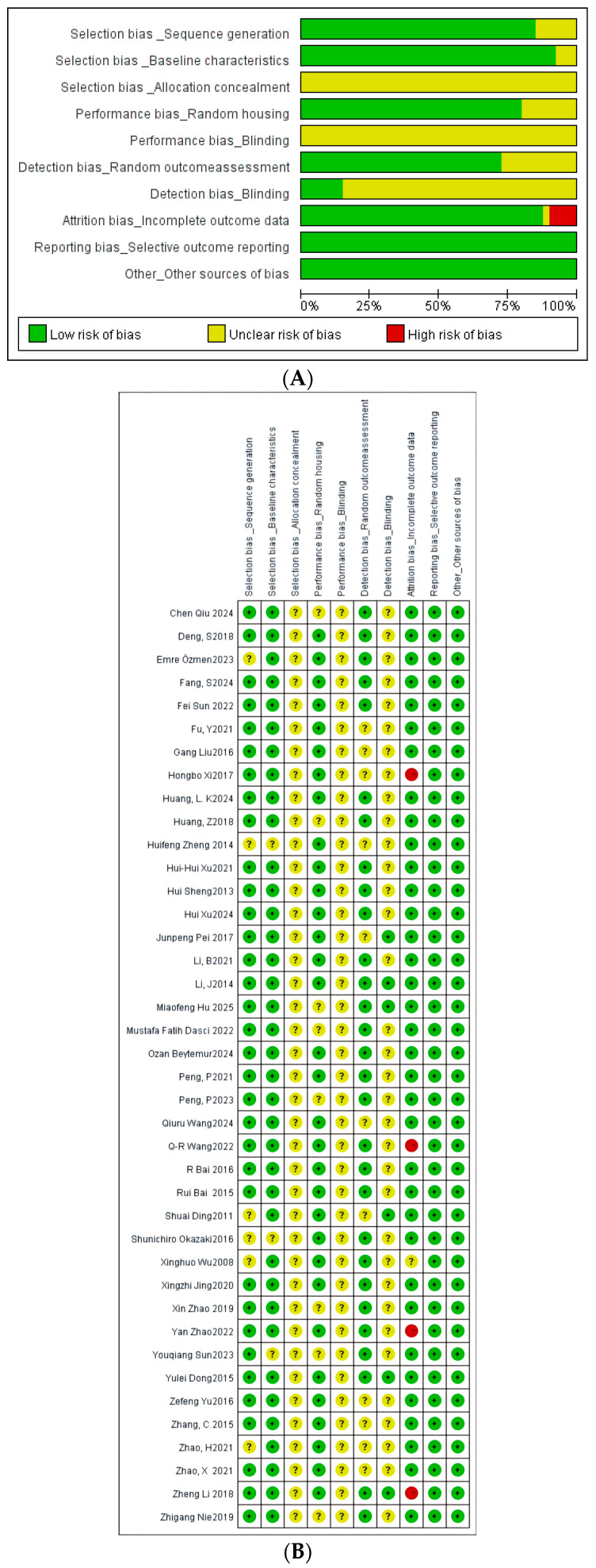
Risk of bias of the included studies. (**A**) Graph of the risk-of-bias. (**B**) Summary of the risk of bias [12,13,14,15,16,17,18,19,20,21,22,23,24,25,26,27,28,29,30,31,32,33,34,35,36,37,38,39,40,41,42,43,44,45,46,47,48,49,50,51].

The results of the risk-of-bias assessments for the included animal studies, evaluated using the SYRCLE tool, are summarized in Figure 3A,B. Selection bias included random sequence generation, baseline characteristics, and allocation concealment; performance bias included random housing and blinding of caregivers administering interventions; detection bias included random outcome assessment and blinding of outcome assessors; attrition bias referred to incomplete outcome data; reporting bias was defined as selective outcome reporting; and other potential sources of bias were categorized as other types of bias.

None of the included studies fully met all the methodological criteria assessed. With respect to selection bias, 15.0% of the studies (*n* = 6) did not clearly describe the method of random sequence generation, and 7.5% (n = 3) inadequately reported baseline characteristics. Unclear risks of bias identified across studies involved mainly allocation concealment, the blinding of caregivers and/or investigators, and the blinding of outcome assessors. Unclear methods for random outcome assessment were reported in 27.5% of the studies (n = 11), indicating potential detection bias. Ten percent of the studies (n = 4) were judged to have a high risk of attrition bias because of incomplete outcome data.

### 3.4. Meta–Regression and Sensitivity Analysis

**Figure 4:**
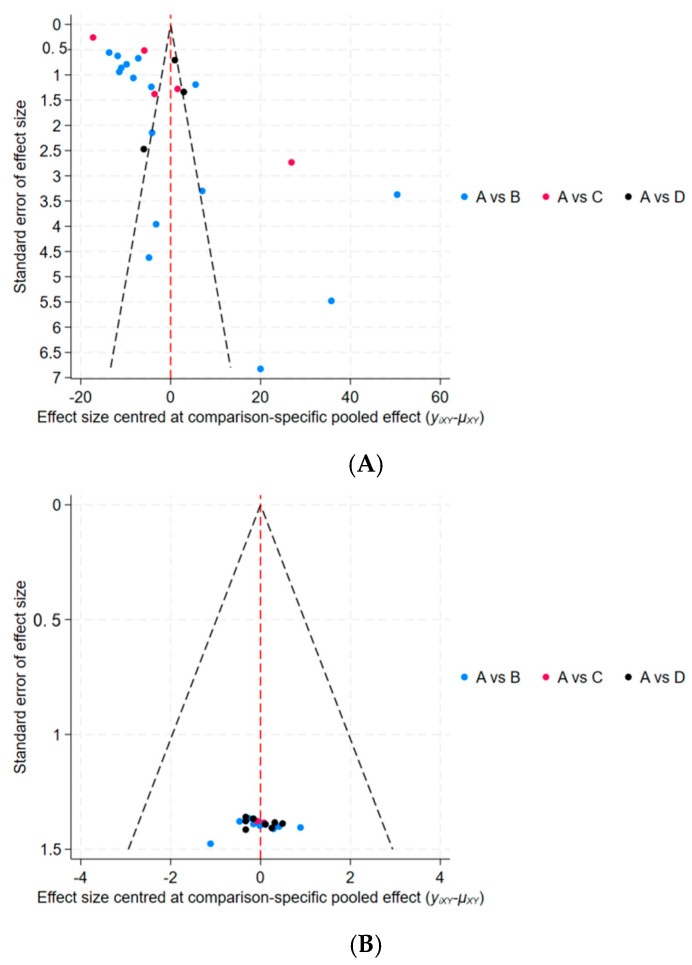
The funnel plots. (**A**) The rate of empty osteocyte lacunae [12,13,15,16,17,19,20,21,23,29,30,31,34,36,37,38,39,44,45,46,48,49,50]. (**B**) The modeling success rate [14,15,16,18,21,22,24,25,26,27,28,32,33,34,35,40,41,42,43,44,46,47,49,50,51].

Meta-regression analysis revealed that neither the species of experimental animals nor the glucocorticoid dosage was a major source of heterogeneity in the present study (both *p* > 0.05). Sensitivity analysis using the leave-one-out method demonstrated that sequential omission of individual studies did not substantially alter the pooled effect sizes, direction of effect, or statistical significance, and all recalculated estimates remained within the original 95% confidence intervals. For the dichotomous outcome of modeling success rate, sensitivity analysis was also performed to assess whether heterogeneity in diagnostic definitions affected the robustness of the pooled results. Sequential exclusion of individual studies, including those using different criteria for defining successful model establishment, did not substantially alter the direction of effect or the overall trend. These findings suggest that the pooled modeling success rate was generally robust, although residual variability caused by non-uniform diagnostic criteria cannot be completely excluded. These findings support the robustness and reliability of the present network meta-analysis. In addition, visual inspection of the funnel plots (Figure 4A,B) indicated that most studies were distributed around the pooled effect estimate, suggesting that substantial publication bias was unlikely overall. However, some comparisons revealed a certain degree of asymmetry, with several studies located relatively far from the central line, indicating the possible presence of small-study effects or potential publication bias. Therefore, although no marked publication bias was observed overall, it cannot be completely excluded. This may be attributable to the preferential publication of positive findings, relatively small sample sizes in some included studies, and a certain degree of between-study heterogeneity.

### 3.5. Meta-Analysis

#### 3.5.1. Results of the Network Meta-Analysis

**Figure 5:**
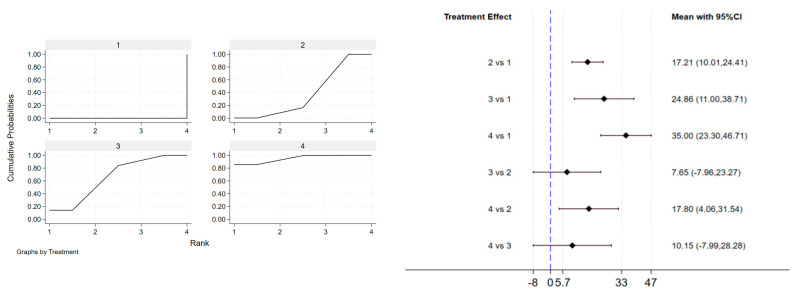
Pairwise comparison forest plots and SUCRA plots of the rate of bone defects in models induced by different doses of lipopolysaccharide combined with glucocorticoids [12,13,15,16,17,19,20,21,23,29,30,31,34,36,37,38,39,44,45,46,48,49,50].

Using the normal control group as a common reference, indirect comparisons were performed among three LPS-plus-glucocorticoid modeling protocols in 31 studies reporting the empty lacunae rate. A random-effects consistency model for continuous variables was applied in the network meta-analysis, as illustrated in Figure 5. The evidence network for the empty lacunae rate did not contain closed loops; therefore, loop-specific local inconsistency testing was not applicable. Accordingly, the random-effects consistency model was retained for the analysis. Comparative analysis demonstrated that all three LPS-plus-glucocorticoid dosing regimens significantly increased the number of empty osteocyte lacunae in the femoral head in the SONFH animal models (all *p* < 0.001). Specifically, the pooled effect size was [*MD* = 35.00, 95% *CI* (23.30, 46.71)] for high-dose LPS plus glucocorticoids, [*MD* = 24.86, 95% *CI* (11.00, 38.71)] for medium-dose LPS plus glucocorticoids, and [*MD* = 17.21, 95% *CI* (10.01, 24.41)] for low-dose LPS plus glucocorticoids. Between-group comparisons revealed a statistically significant difference between high-dose and low-dose LPS regimens [*MD* = 17.80, 95% *CI* (4.06, 31.54), *p* < 0.05], whereas no significant differences were observed in other pairwise comparisons (all *p* > 0.05). Ranking based on SUCRA probabilities indicated that high-dose LPS combined with glucocorticoids had the highest likelihood of achieving the greatest empty lacunae rate (85.5%), followed by low-dose LPS (83.5%) and medium-dose LPS (69.8%). This ranking merely reflects the relative trend of effect magnitudes and does not indicate statistically significant superiority among groups.

**Figure 6:**
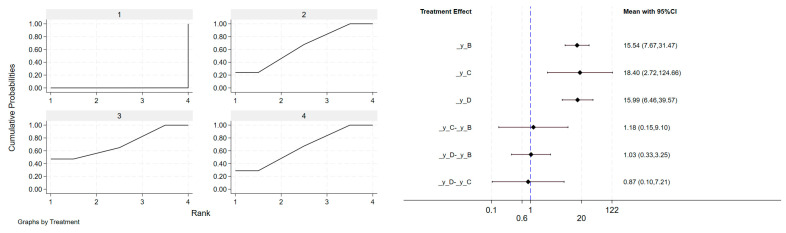
Pairwise comparison forest plots and SUCRA plots of the modeling success rates for different doses of lipopolysaccharide combined with glucocorticoids [14,15,16,18,21,22,24,25,26,27,28,32,33,34,35,40,41,42,43,44,46,47,49,50,51].

Using the normal control group as a common reference, indirect comparisons were conducted among three LPS-plus-glucocorticoid modeling protocols in 26 studies reporting modeling success rates. A random-effects consistency model for dichotomous variables was used in the network meta-analysis, as shown in Figure 6. Similarly, the evidence network for the modeling success rate contained no closed loops; therefore, loop-specific local inconsistency testing was not applicable, and the random-effects consistency model was retained. All three LPS-plus-glucocorticoid dosing regimens significantly improved the modeling success rate in the animal models compared with that in the normal control group (*p* < 0.05). The pooled effect sizes were as follows: high-dose LPS plus glucocorticoids [*RR* = 15.99, 95% *CI* (6.46, 39.57)], medium-dose LPS plus glucocorticoids [*RR* = 18.40, 95% *CI* (2.72, 124.66)], and low-dose LPS plus glucocorticoids [*RR* = 15.54, 95% *CI* (7.67, 31.47)]. Probability ranking analysis revealed that medium-dose LPS combined with glucocorticoids was the most effective regimen for modeling success (48.0%), followed by high-dose LPS (27.9%) and low-dose LPS (24.1%). This probability ranking indicates only a trend difference without statistically significant superiority. Therefore, it cannot be concluded that medium-doses of LPS yield a significantly higher modeling success rate than high- or low-doses of LPS do.

#### 3.5.2. Subgroup Analysis of Different Influencing Factors

Given the substantial differences among the three dosing protocols and statistically significant between-subgroup heterogeneity, subgroup analyses stratified by animal species, sex, glucocorticoid dosage, and modeling duration were performed separately for studies evaluating the empty lacunae rate under each of the three regimens.

**Figure 7:**
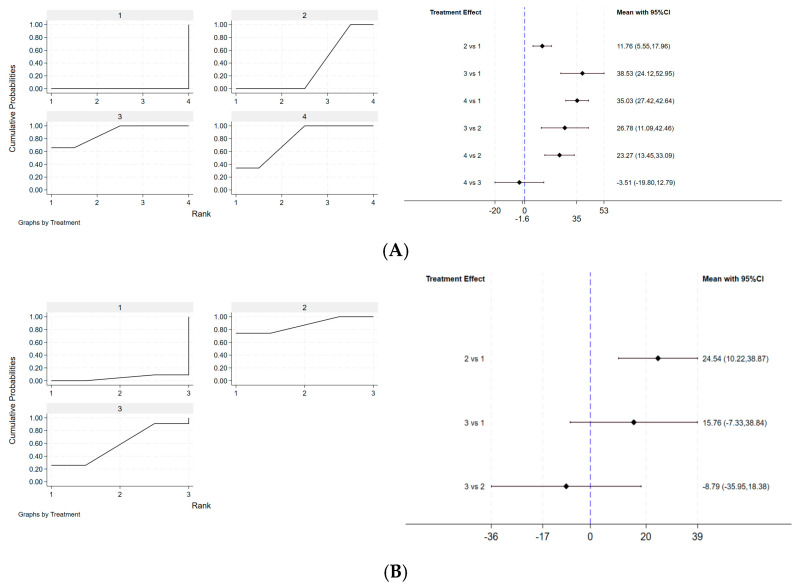
Pairwise comparison forest plots and SUCRA plots for the rat subgroup (**A**) [12,16,19,20,29,30,31,34,38,39,44,45,46,48,49,50] and the rabbit subgroup (**B**) [13,15,17,21,23,36,37].

With the normal control group as the reference, subgroup analyses by animal species (rabbit and rat) were conducted, as illustrated in Figure 7A,B. The rat subgroup included 20 studies [12,16,19,20,29,30,31,34,38,39,44,45,46,48,49,50]: 11 in the low-dose LPS group, 2 in the medium-dose group, and 7 in the high-dose group. The pooled effect sizes were as follows: low-dose LPS [*MD* = 11.76, 95% *CI* (5.55, 17.96)], medium-dose LPS [*MD* = 38.53, 95% *CI* (24.12, 52.95)], and high-dose LPS [*MD* = 35.03, 95% *CI* (27.42, 42.64)]. The empty lacunae rate increased significantly with increasing LPS dosage, demonstrating a clear dose–response relationship. The SUCRA ranking suggested that medium-dose LPS had a relatively higher probability of being the optimal regimen, although this represented only a trend without statistically definitive superiority. The rabbit subgroup included 10 studies [13,15,17,21,23,36,37]: 7 in the low-dose group and 3 in the medium-dose group, with no studies in the high-dose group. The pooled effect sizes were as follows: low-dose LPS (*MD* = 24.54, 95% *CI*: 10.22–38.87) and medium-dose LPS (*MD* = 15.76, 95% *CI*: −7.33–38.84). No significant difference in the empty lacunae rate was observed between low- and medium-doses of LPS. SUCRA analysis indicated that low-dose LPS had a relatively higher probability of being the optimal regimen, which was only a trend without supporting data from high-dose LPS. Marked differences were observed in the dose–response patterns of LPS-plus-steroid-induced osteonecrosis of the femoral head between rabbits and rats, with statistically significant differences between species subgroups.

**Figure 8:**
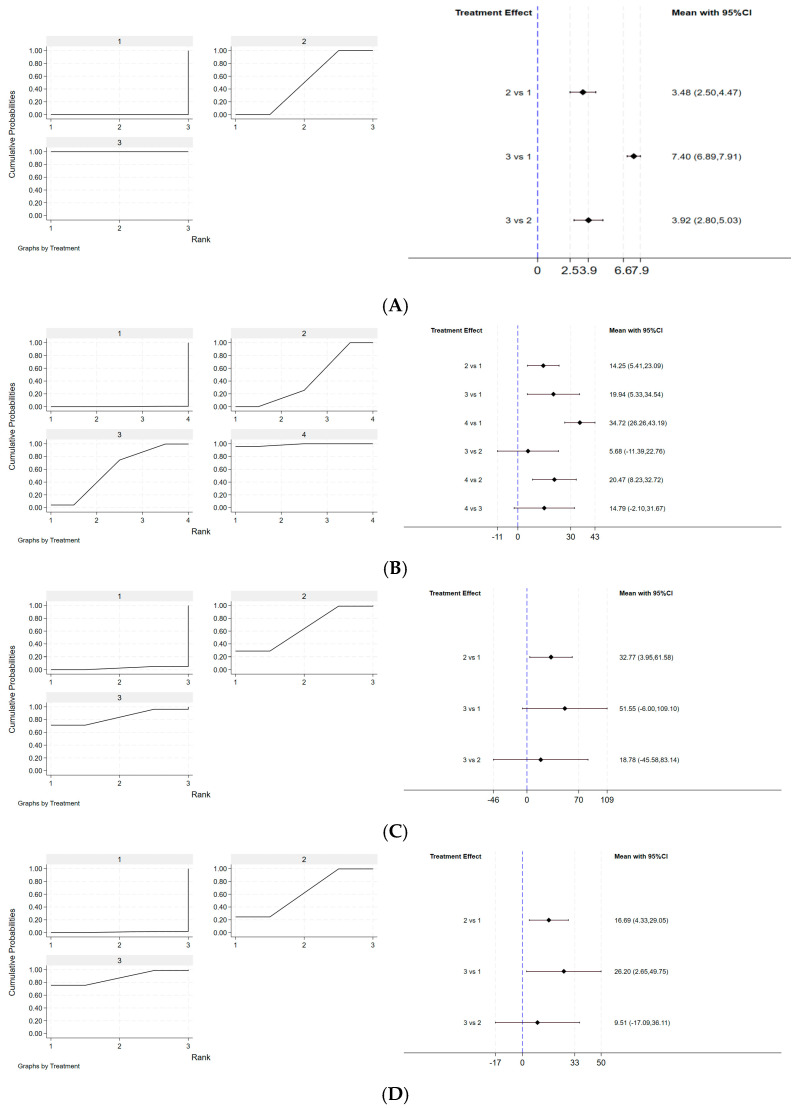
Pairwise comparison forest plots and SUCRA plots for the 2–week (**A**) [19,23,36], 4–week (**B**) [12,16,19,20,23,30,34,36,37,44,45,46,49,50], 6–week (**C**) [15,21,29,38], and 8–week (**D**) [13,17,19,23,39] subgroups.

Using the normal control group as a common reference, network meta-analysis was performed in subgroups stratified by modeling duration at 2, 4, 6, and 8 weeks, as shown in Figure 8A–D. The 2-week subgroup included 3 studies [19,23,36]: 2 in the low-dose LPS group and 1 in the medium-dose group, with no high-dose studies. Compared with that in the control group, the empty lacunae rate was significantly greater in both the low-dose and medium-dose groups [*MD* = 3.48, 95% *CI*: 2.50–4.47; *MD* = 7.40, 95% *CI*: 6.89–7.91]. Moreover, the effect in the medium-dose group was significantly greater than that in the low-dose group [*MD* = 3.92, 95% *CI*: 2.80–5.03], indicating a clear dose–response effect at the early stage. The 4-week subgroup included 14 studies [12,16,19,20,23,30,34,36,37,44,45,46,49,50]: 6 in the low-dose LPS group, 2 in the medium-dose group, and 6 in the high-dose group. The empty lacunae rate was significantly higher in the low-, medium-, and high-dose groups than in the control group [*MD* = 14.25, 95% *CI*: 5.41–23.09; *MD* = 19.94, 95% *CI*: 5.33–34.54; *MD* = 34.72, 95% *CI*: 26.26–43.19]. Compared with the low-dose group, the high-dose group had significantly greater effects [*MD* = 20.47, 95% *CI*: 8.23–32.72], whereas no significant differences were observed between the medium-dose group and either the low- or high-dose groups. SUCRA ranking revealed an obvious dose–response effect for the empty lacunae rate at 2–4 weeks, with the optimal dose at different time points representing only a probabilistic trend. The 6-week subgroup included 4 studies [15,21,29,38]: 3 in the low-dose LPS group, 1 in the medium-dose group, with no high-dose studies. Compared with the control group, only the low-dose group presented a significantly greater number of empty lacunae [*MD* = 32.77, 95% *CI*: 3.95–61.58]. No significant differences were detected between the medium-dose group and either the control or low-dose groups, and the dose–response effect disappeared. The 8-week subgroup included 5 studies [13,17,19,23,39]: 4 in the low-dose LPS group and 1 in the medium-dose group, with no high-dose studies. Compared with the control group, both the low- and medium-dose groups presented significantly higher empty lacunae rates [*MD* = 16.69, 95% *CI*: 4.33–29.05; *MD* = 26.20, 95% *CI*: 2.65–49.75], with no significant difference between the two treatment groups, indicating stable model establishment. Cumulative ranking probability plots suggested that the dose–response effect diminished at 6–8 weeks, with no statistically significant differences among groups and modeling conditions tending to stabilize without a definitively optimal dose. Between-subgroup comparisons demonstrated marked differences in dose–response patterns across different modeling durations, with a clear temporal relationship and statistically significant subgroup effects.

**Figure 9:**
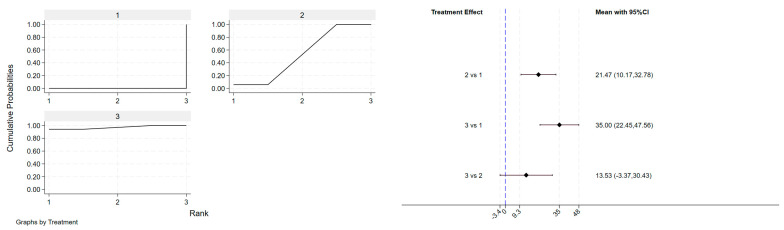
Pairwise comparison forest plot and SUCRA plot for the male subgroup [12,15,16,17,19,20,30,34,44,45,46,48,49,50].

Using the normal control group as the common reference, subgroup analysis was performed according to the sex of the experimental animals, as illustrated in Figure 9. Because of the extremely limited number of included studies involving female animals, a valid network meta-analysis could not be conducted in the female subgroup; therefore, the analysis was performed only in the male subgroup. The male subgroup comprised 9 studies in the low-dose LPS group [15,16,17,19,20,30,34] and 7 studies in the high-dose LPS group [12,44,45,46,48,49,50], with no studies in the medium-dose LPS group. Compared with that in the normal control group, the empty lacunae rate significantly increased in both the low-dose and high-dose LPS groups [*MD* = 21.47, 95% *CI*: 10.17–32.78; *MD* = 35.00, 95% *CI*: 22.45–47.56]. Although the empty lacunae rate was numerically greater in the high-dose LPS group than in the low-dose group, the difference was not statistically significant [*MD* = 13.53, 95% *CI*: −3.37–30.43]. Cumulative ranking probability plots indicated that the high-dose LPS regimen was associated with a numerically higher empty lacunae rate and a relatively higher probability of being the optimal protocol, but the difference was not statistically significant compared with the low-dose regimen.

**Figure 10:**
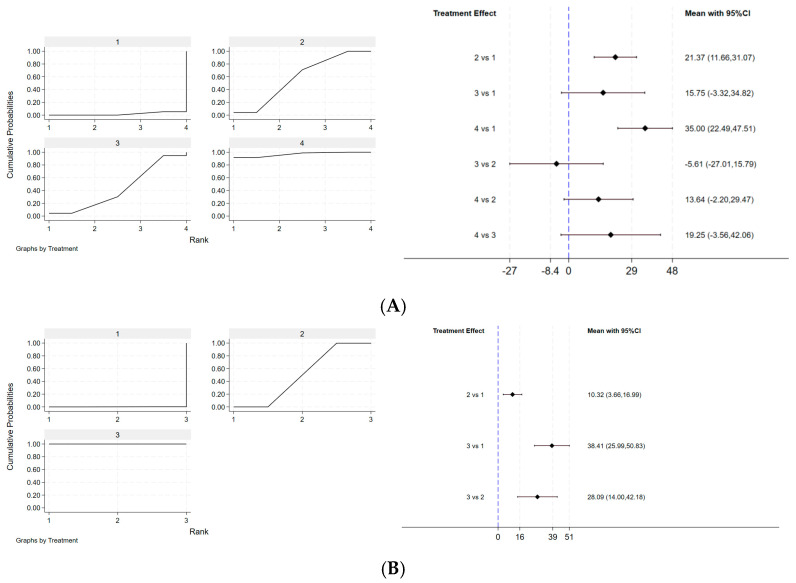
Pairwise comparison forest plots and SUCRA plots for the low-dose glucocorticoid (**A**) [12,13,15,16,17,19,21,23,36,37,44,45,46,48,49,50] and high-dose glucocorticoid (**B**) [20,29,30,31,34,38,39] subgroups.

Using the normal control group as a common reference, subgroup analysis was conducted according to glucocorticoid dosage, as shown in Figure 10A,B. After conversion to clinically equivalent doses, the included studies were divided into a low-dose glucocorticoid subgroup (<120 mg/kg) and a high-dose glucocorticoid subgroup (≥120 mg/kg). The low-dose glucocorticoid subgroup included 22 studies [12,13,15,16,17,19,21,23,36,37,44,45,46,48,49,50]: 12 in the low-dose LPS group, 3 in the medium-dose group, and 7 in the high-dose group. Compared with that in the normal control group, the empty lacunae rate was significantly greater in both the low-dose and high-dose LPS groups [*MD* = 21.37, 95% *CI*: 11.66–31.07; *MD* = 35.00, 95% *CI*: 22.49–47.51], whereas no significant difference was observed between the medium-dose LPS group and the control group [*MD* = 15.75, 95% *CI*: −3.32–34.82]. No statistically significant differences were found among the three LPS dose subgroups. The high-dose glucocorticoid subgroup included 9 studies [20,29,30,31,34,38,39]: 7 in the low-dose LPS group and 2 in the medium-dose group, with no high-dose LPS studies. Compared with the normal control group, both the low-dose and medium-dose LPS groups presented significantly higher empty lacunae rates [*MD* = 10.32, 95% *CI*: 3.66–16.99; *MD* = 38.41, 95% *CI*: 25.99–50.83]. Furthermore, the number of empty lacunae was significantly greater in the medium-dose LPS group than in the low-dose group [*MD* = 28.09, 95% *CI*: 14.00–42.18].

Between-subgroup comparisons revealed a significant interaction between glucocorticoid dosage and LPS efficacy: the dose–response relationship of LPS was markedly enhanced under a high-dose glucocorticoid background. In the high-dose glucocorticoid treatment group, the number of empty lacunae was significantly greater in the medium-dose LPS group than in the low-dose group (*p* < 0.05). In the low-dose glucocorticoid setting, high-dose LPS showed a favorable trend but was not statistically definitively superior.

## 4. Discussion

The core mechanism underlying LPS plus GC-induced steroid-induced osteonecrosis of the femoral head lies in their synergistic effects [52,53]. LPS activates systemic inflammatory pathways, triggers local inflammatory infiltration in the femoral head, and impairs vascular endothelial function [54]. At the molecular level, this process is closely linked to activation of the TLR4/NF-κB axis and increased release of inflammatory mediators such as TNF-alpha, IL-1beta, and IL-6, which aggravate endothelial damage, thrombosis, and marrow microcirculatory disturbance. In contrast, GCs disrupt normal bone metabolic homeostasis by suppressing osteoblast activity and promoting osteocyte apoptosis [52]. Our findings suggest that different LPS dosing strategies lead to varying degrees of osteonecrotic change and mortality risk, highlighting the need to balance modeling efficacy with animal safety. Although some differences in rankings were observed, most pairwise comparisons among dose groups were not statistically significant. Therefore, *SUCRA* probability rankings should be interpreted as indicating relative trends rather than definitive evidence for a single optimal regimen. The selection of a modeling protocol should be comprehensively determined on the basis of statistical significance, effect trends, and animal safety. Different LPS and GC dose combinations not only affect modeling efficacy but also influence animal mortality, which further affects pathological phenotype matching, experimental reproducibility, and practical applicability. This represents a critical issue in the standardization of SONFH modeling: how to achieve satisfactory modeling outcomes while controlling animal mortality and improving model reliability [53].

High-dose LPS can enhance modeling efficacy by producing higher empty lacunae rates and more pronounced pathological changes. However, this advantage is accompanied by increased systemic toxicity and elevated mortality, which restricts its widespread application [54]. Therefore, researchers should carefully balance modeling efficiency with animal welfare when selecting LPS-GC protocols. Medium-dose LPS, particularly under a high-dose GC background, may achieve reproducible osteonecrotic lesions while reducing mortality, offering a compromise between pathological intensity and animal survival. Low-dose LPS may be appropriate in endotoxin-sensitive species or for rapid screening studies, where minimizing mortality is a priority. Explicitly weighing efficacy against safety may help establish standardized, humane, and reproducible SONFH experimental protocols [55].

Given that modeling efficacy is simultaneously regulated by multiple factors, including animal species, sex, GC dosage, and modeling duration, single-dose comparisons are insufficient to fully elucidate modeling patterns or provide precise model selection criteria for diverse research objectives. Therefore, protocol selection should consider not only the magnitude of pathological changes but also the intended study purpose: stronger induction strategies may be useful for mechanistic studies requiring pronounced osteonecrotic lesions, whereas regimens with lower toxicity and better survival may be preferable for long-term intervention or safety-oriented studies. Combined with recent evidence from related studies, these considerations may provide more practical evidence-based support for the standardized establishment of SONFH animal models and subsequent preclinical research.

### 4.1. Analysis of the Influence of Experimental Animal Species

The present study revealed distinct dose–response trends between rats and rabbits in the LPS-GC-induced SONFH model. This divergence may be explained by interspecies differences in innate immune responsiveness, endotoxin sensitivity, bone remodeling characteristics, and GC-related skeletal injury. In the rat subgroup, the empty lacunae rate increased with increasing LPS dose, suggesting that rats exhibit a relatively graded response to LPS-induced inflammation and GC-mediated bone injury. This may be related to the broader dose tolerance of rats to endotoxin stimulation, allowing LPS escalation to produce progressively stronger inflammatory and vascular injury before reaching excessive systemic toxicity. In contrast, the rabbit subgroup did not show a clear LPS dose–response relationship, and high-dose LPS data were unavailable. A plausible explanation is that rabbits are more sensitive to endotoxin than rodents: experimental studies have shown that rabbits can develop strong TNF-α-mediated inflammatory responses at substantially lower LPS doses than small rodents, and low-dose LPS alone has been reported to induce osteonecrotic changes in rabbits [56,57,58]. Therefore, in rabbits, low-dose LPS may already be sufficient to trigger inflammatory endothelial injury and microcirculatory disturbance, whereas further dose escalation may not proportionally increase osteonecrotic severity but may instead increase systemic toxicity and mortality risk.

Species-specific bone biology may also contribute to these findings. Rats are characterized by rapid bone turnover and high experimental reproducibility, making them suitable for observing dose-dependent pathological progression and for long-term mechanistic or interventional studies [59,60]. Rabbits, although widely used in orthopedic and bone-regeneration research, differ from rodents in skeletal maturity, cortical remodeling, and bone microarchitecture; these differences may alter the development, repair, and stabilization of osteonecrotic lesions [61,62]. In addition, GC pharmacokinetics and GC-induced skeletal responses may vary across species, which could influence the interaction between LPS-induced inflammation and GC-mediated osteocyte apoptosis, adipogenic differentiation, and vascular impairment. These differences suggest that rats and rabbits should not be regarded as interchangeable SONFH models. Rats may be preferable when the research aim is to examine LPS dose–response patterns, molecular mechanisms, or therapeutic interventions under relatively reproducible conditions. Rabbits may be more appropriate for rapid model establishment or preliminary drug screening, especially when low-dose LPS can induce sufficient pathology; however, the lack of high-dose LPS evidence in rabbits and their potential endotoxin sensitivity require cautious dose escalation and close mortality monitoring. Future studies should directly compare species-specific immune, vascular, bone-remodeling, and GC-metabolic responses under standardized LPS-GC protocols to improve model selection, reproducibility, and translational relevance.

### 4.2. Analysis of Factors Influencing the Modeling Period

On the basis of the subgroup analysis of modeling duration in our network meta-analysis, we clarified the complete temporal evolution pattern of the SONFH model: Weeks 2–4 represented the progressive stage of bone injury, during which the dose–response effect was significant, whereas the optimal dose at different time points only indicated a trend. Weeks 6–8 constitute the stable stage of modeling, where the dose–response effect disappears, and low-dose LPS alone is sufficient to maintain a stable osteonecrotic state. This temporal relationship is highly consistent with the pathological progression of SONFH [63,64] and provides a reference for selecting modeling durations according to different research purposes.

In the early stage of modeling (weeks 2–4), the synergistic effect of LPS combined with GC gradually emerges, with progressive inflammatory activation, vascular impairment, osteocyte apoptosis, and increasing empty lacunae rates [18,65,66]. Therefore, this period may be more suitable for early mechanistic studies requiring pronounced pathological changes, such as inflammation-, endothelial injury-, oxidative stress-, or apoptosis-focused investigations. In contrast, during weeks 6–8, osteonecrotic lesions tend to stabilize and the LPS dose–response effect gradually diminishes [67]. Thus, for long-term intervention, therapeutic evaluation, or safety-oriented studies, a 6–8-week modeling period may be preferable because it prioritizes model stability and animal survival over maximal pathological severity. Nevertheless, these temporal patterns should be interpreted as purpose-oriented guidance rather than evidence for a single optimal duration or dose.

### 4.3. Analysis of the Influence of Animal Sex

In the network meta-analysis of the present study, only male animals could be included in the subgroup analysis because of the extremely limited number of eligible studies involving female animals. The results revealed that in male animals, both low- and high-doses of LPS significantly increased the empty lacunae rate. Although high-doses of LPS tended to increase the likelihood of being the optimal modeling regimen, the difference was not statistically significant. These findings are highly consistent with recent research trends worldwide, and their underlying mechanism is closely related to the osteoprotective effects of endogenous estrogen [68,69], revealing a notable sex-related data gap in current SONFH modeling studies.

Endogenous estrogen in female animals protected bone tissue through multiple pathways. On the one hand, estrogen inhibits osteocyte apoptosis, reduces oxidative stress injury, and maintains normal bone metabolism [59]. On the other hand, estrogen improves the blood supply to the femoral head, alleviates vascular endothelial injury and thrombosis, and attenuates LPS-induced inflammatory responses, thereby lowering the incidence of osteonecrosis [70]. This protective effect significantly increases the difficulty of model establishment in females, resulting in a much lower modeling success rate in females than in males. Consequently, studies using female animals to model SONFH are extremely rare globally, making it difficult to form robust evidence-based conclusions. Therefore, at the present stage, male animals remain the only reliable choice for establishing SONFH animal models, providing clear evidence-based support for model selection in subsequent research.

### 4.4. Analysis of Factors Influencing Glucocorticoid Dosage

Glucocorticoid dosage is a key factor regulating the efficacy of LPS-combined modeling. After conversion to equivalent doses, all dosages were standardized to methylprednisolone equivalents. Under a background of high-dose methylprednisolone (≥120 mg/kg equivalent), the dose–response relationship of LPS was significantly enhanced, and medium-dose LPS produced a significantly stronger modeling effect. In contrast, under low-dose methylprednisolone treatment, the dose–response effect of LPS was markedly attenuated; high-dose LPS tended to be more favorable, whereas medium-dose LPS did not significantly differ from that in the normal control group.

This discrepancy arises primarily from the synergistic osteonecrotic effect between methylprednisolone and LPS. High-dose methylprednisolone amplifies LPS-induced osteonecrosis through multiple pathways: it directly induces osteocyte apoptosis, suppresses osteoblast activity, and disrupts normal bone architecture [71]; it also impairs vascular endothelial function and compromises femoral head perfusion [72]; and it synergizes with LPS to exaggerate inflammatory responses and promote inflammatory cytokine release, thereby exacerbating bone injury. Low-dose methylprednisolone exerts relatively mild damaging effects on bone tissue and cannot effectively synergize with LPS to induce sufficient osteonecrosis. High doses of LPS may therefore be required to overcome bony defense mechanisms and achieve stable modeling, but such doses frequently trigger severe systemic inflammatory toxicity and increase animal mortality. Accordingly, high-dose methylprednisolone combined with medium-dose LPS may be considered a relatively balanced option between modeling efficacy and safety, rather than a definitively optimal protocol.

Elevated mortality associated with high-dose LPS represents a critical issue that must be carefully controlled in preclinical experiments, as endotoxin-induced lethality is strongly dose dependent and closely associated with systemic inflammatory responses [73]. Therefore, LPS dose should be selected in relation to both the GC background dose and the intended research objective. For mechanistic studies requiring strong pathological stimulation, such as studies of acute inflammation, endothelial dysfunction, or apoptosis-related signaling, high-dose LPS or medium-dose LPS under a high-dose GC background may be considered because these regimens can induce more evident osteonecrotic changes. However, this strategy should be used cautiously because excessive LPS exposure may increase systemic toxicity and mortality.

For studies emphasizing long-term observation, therapeutic efficacy, or safety evaluation, the modeling strategy should shift from maximizing pathological severity to improving model stability and animal survival. In this context, medium-dose LPS under a high-dose GC background may provide a more balanced inflammatory–apoptotic–vascular phenotype, whereas low-dose LPS may be preferable in endotoxin-sensitive species such as rabbits or in rapid screening experiments. On the basis of the present findings, three practical strategies may help reduce mortality while maintaining acceptable modeling efficacy: avoiding high-dose LPS in sensitive species, preferring medium-dose LPS when combined with high-dose GC, and extending the observation period to 6–8 weeks when stable osteonecrotic lesions are required. Future optimization may involve fractionated administration, reduced single doses, and extended dosing intervals to improve animal survival while maintaining sufficient pathological features for model evaluation [74].

### 4.5. Molecular-Pathway Interpretation of LPS-Glucocorticoid SONFH Models

The empty osteocyte lacunae rate represents a downstream histopathological consequence of multiple upstream molecular events, including inflammatory cytokine release, endothelial injury, oxidative stress, programmed cell death, adipogenic differentiation, and impaired osteogenic and angiogenic repair [75]. In this context, different LPS doses may generate distinct molecular phenotypes rather than merely producing different degrees of osteonecrosis.

Mechanistically, LPS acts primarily through innate immune activation. By stimulating the TLR4/NF-κB signaling pathway, LPS promotes the release of TNF-α, IL-1β, IL-6, and other inflammatory mediators, thereby enhancing osteoclastogenesis, endothelial activation, coagulation imbalance, and microvascular dysfunction in the femoral head [76]. This mechanism may explain why higher LPS exposure was associated with increased empty lacunae rates, particularly during the early modeling period, when inflammatory amplification is expected to be most prominent. In parallel, glucocorticoids provide a permissive osteonecrotic background by disrupting bone-cell survival, osteogenic differentiation, and microvascular integrity. High-dose glucocorticoids can induce osteocyte and osteoblast apoptosis through mitochondrial dysfunction, oxidative stress, and caspase-dependent pathways; suppress RUNX2- and BMP-mediated osteogenesis; and promote PPARγ-dependent adipogenic differentiation of bone marrow mesenchymal stem cells [77]. Therefore, the interaction between glucocorticoid dosage and LPS efficacy observed in the subgroup analysis is biologically plausible. Under a high-glucocorticoid background, a medium-dose LPS stimulus may be sufficient to drive the model toward a stable osteonecrotic phenotype without requiring excessive endotoxin exposure. Vascular and angiogenic impairment further links the animal model to the clinical pathophysiology of SONFH. LPS-induced endothelial inflammation and glucocorticoid-induced endothelial dysfunction may converge on reduced nitric oxide bioavailability, microthrombus formation, vascular rarefaction, and impaired VEGF/eNOS-mediated angiogenic repair [78]. These molecular events may underlie the transition from early inflammatory injury to relatively stable osteonecrosis at 6–8 weeks. At later stages, the disappearance of a clear LPS dose–response relationship may indicate that downstream vascular occlusion and bone-cell death have reached a plateau, making additional endotoxin exposure less informative and potentially more harmful.

Taken together, the modeling hierarchy observed in this study suggests that different LPS-GC combinations may correspond to distinct molecular–pathological phenotypes and should therefore be selected according to the research objective. High-dose LPS emphasizes acute inflammatory and endothelial injury pathways but is accompanied by increased mortality, making it more suitable for short-term mechanistic exploration rather than long-term safety evaluation. Medium-dose LPS under a high-GC background may provide a more balanced inflammatory–apoptotic–vascular phenotype and may be more appropriate for interventional or therapeutic evaluation. Low-dose LPS may be adequate in endotoxin-sensitive species or rapid screening models. Future SONFH modeling studies should incorporate standardized molecular endpoints, including TLR4/NF-κB activation, TNF-α/IL-1β/IL-6 levels, apoptosis or programmed cell death markers, oxidative-stress indicators, PPARγ/RUNX2 expression, VEGF/eNOS signaling, and thrombosis-related parameters [79]. Incorporating these molecular outcomes would substantially enhance model standardization, mechanistic interpretability, and translational relevance for future preclinical and network meta-analytic studies.

### 4.6. Clinical Relevance and Translational Considerations

While the LPS plus glucocorticoid protocol effectively induces SONFH in animals, several limitations restrict the direct translation of these findings to humans. First, human SONFH typically develops gradually under chronic glucocorticoid exposure combined with systemic metabolic disturbances, including lipid disorders, endothelial dysfunction, and impaired bone remodeling [80], whereas the LPS-based animal models introduce an acute inflammatory insult that accelerates osteonecrosis. This difference in disease kinetics and multifactorial etiology limits the model’s ability to fully replicate the progressive pathophysiology observed clinically. Second, interspecies variations in bone microarchitecture, immune response, and glucocorticoid metabolism affect the severity and pattern of osteonecrotic lesions, which may not entirely correspond to human pathology [81]. Third, the use of young, healthy animals fails to account for comorbidities commonly present in patients, such as diabetes or dyslipidemia, which can significantly influence disease progression and therapeutic response. Consequently, while these models provide valuable mechanistic insights—such as inflammatory activation, endothelial injury, osteocyte apoptosis, and impaired angiogenic-osteogenic coupling—they have limited predictive value for clinical outcomes. Therefore, caution is warranted when extrapolating preclinical findings to humans. Future studies should aim to integrate chronic glucocorticoid exposure, metabolic comorbidities, and vascular remodeling factors to enhance the translational relevance and clinical applicability of SONFH animal models.

In addition, larger animal models should be considered in future SONFH research to improve translational relevance. Compared with rodents and rabbits, animals such as sheep, goats, pigs, or dogs have femoral head size, joint loading patterns, bone remodeling characteristics, and surgical accessibility that are closer to those of humans [82,83]. Therefore, they may be more suitable for evaluating late-stage structural collapse, biomechanical changes, imaging progression, and surgical or implant-based interventions. However, several barriers currently limit their widespread application, including substantially higher costs, longer experimental periods, specialized housing facilities, stricter ethical review, and limited sample sizes [84]. Moreover, standardized protocols for inducing reproducible SONFH in large animals remain insufficiently established, and interspecies differences in sensitivity to LPS and glucocorticoids may complicate dose conversion, toxicity control, and mortality reduction [65]. Thus, although large-animal models have important translational potential, standardized, safe, and reproducible modeling protocols should be established before their broader use in preclinical SONFH research.

### 4.7. Strengths and Limitations

This study employed a network meta-analysis design to systematically synthesize the current research evidence on animal models of steroid-induced osteonecrosis of the femoral head established by lipopolysaccharide combined with glucocorticoids. Through comprehensive comparisons of multiple dosing regimens and influencing factors, relatively robust analytical conclusions were derived, which can provide reliable evidence-based support for the standardized establishment of this model. First, by systematically stratifying low-, medium-, and high-dose LPS combined with glucocorticoid protocols and conducting multidimensional subgroup analyses on the basis of animal species, modeling duration, glucocorticoid dosage, and sex, this study clearly revealed the variation patterns of modeling efficacy under different conditions [85]. Moreover, the trade-off between modeling efficiency and animal safety was taken into account, effectively addressing the limitations of previous similar studies that performed only pairwise comparisons, lacked dose stratification, and failed to conduct integrated multifactor analysis. Second, by unifying the conversion standard for equivalent glucocorticoid doses, heterogeneity caused by inconsistent dosage expression across studies was reduced to a certain extent, rendering comparisons between different modeling protocols more objective and credible. These findings provide a directly applicable practical reference for protocol design and dose selection in subsequent similar animal experiments.

Nevertheless, this study has several limitations. First, objective differences existed among the included literature in terms of animal strains, LPS administration routes, and specific glucocorticoid formulations. Although adjusted by equivalent dose conversion and subgroup analyses, inter–study heterogeneity could not be completely eliminated. Second, the number of studies focusing on high-dose LPS in rabbit models was relatively small, and the stability and generalizability of the relevant findings require verification by further research. Third, although no language restrictions were applied in the electronic database search and the complete search strategies for Embase, PubMed, and Web of Science were provided as Supplementary Material, unpublished grey literature and manual searches of additional sources were not fully conducted, which may still introduce potential publication or retrieval bias. Fourth, this study used the empty lacunae rate as the primary outcome measure and did not quantitatively synthesize molecular endpoints such as inflammatory cytokines, TLR4/NF-κB activation, apoptosis markers, oxidative-stress indicators, angiogenesis-related proteins, lipid-metabolism markers, or hemodynamic parameters. This limits the ability to directly compare molecular phenotypes among modeling protocols and leaves room for improvement in the comprehensive assessment of pathological characteristics of the model. Fifth, most dose groups showed no statistically significant differences, and only trend-based judgments could be made according to the cumulative ranking probability; thus, a single and definitive optimal modeling protocol could not be identified. The corresponding recommendations provide only evidence-based references for model selection and need to be further validated and refined in future studies with larger sample sizes and more standardized experimental designs. Sixth, this study constructed only the core framework of the standard operating procedure on the basis of existing data and did not complete multicenter validation or full workflow development; hence, large-sample and standardized experiments are still needed for further optimization in the future.

## 5. Conclusions

The modeling efficacy of LPS plus steroid-induced SONFH animal models is jointly regulated by animal species, LPS dose, glucocorticoid dose, modeling duration, and the molecular phenotype being targeted. No statistically significant differences were observed among most dose groups, and the SUCRA probability ranking only indicated trends without supporting a definitive optimal protocol. High-dose LPS can increase the empty lacunae rate and may be suitable for acute inflammation- and endothelial-injury-focused mechanistic studies, but it is associated with elevated animal mortality. Medium-dose LPS, particularly under a high-dose glucocorticoid background, may better balance TLR4/NF-κB inflammatory activation, steroid-induced apoptosis/adipogenesis, vascular injury, and animal safety. Low-dose LPS may be appropriate in endotoxin-sensitive species such as rabbits or in rapid screening models. Accordingly, the following recommendations are proposed on the basis of trend analysis, safety considerations, and molecular relevance: for long-term mechanistic investigations or interventional studies, rat models are preferred when using high-dose glucocorticoids combined with medium-dose LPS, with a modeling period of 6–8 weeks; for short-term rapid modeling or drug screening, low-dose LPS is recommended for use in rabbit models, with a modeling period of 2–4 weeks. Future studies should incorporate standardized molecular endpoints, including inflammatory, apoptotic, oxidative-stress, angiogenic, osteogenic, adipogenic, and coagulation-related markers, to improve the molecular interpretability and translational value of SONFH animal models.

## Data Availability

No new data were created or analyzed in this study. Data sharing is not applicable to this review article.

## Supplementary Materials

The following supporting information can be downloaded at: https://www.mdpi.com/article/10.3390/ijms27125416/s1.

## Abbreviations

The following abbreviations are used in this manuscript:

SONFH	steroid-induced osteonecrosis of the femoral head
LPS	lipopolysaccharide
GC	glucocorticoid
PRISMA	Preferred Reporting Items for Systematic Reviews and Meta-Analyses
PROSPERO	International Prospective Register of Systematic Reviews
SYRCLE	Systematic Review Centre for Laboratory Animal Experimentation
SD	standard deviation
MD	mean difference
CI	confidence interval
RR	relative risk
SUCRA	surface under the cumulative ranking curve
